# Psychological problems of cancer patients: a cancer distress screening with a cancer-specific questionnaire

**DOI:** 10.1038/sj.bjc.6601986

**Published:** 2004-07-06

**Authors:** P Herschbach, M Keller, L Knight, T Brandl, B Huber, G Henrich, B Marten-Mittag

**Affiliations:** 1 Institute and Outpatient Clinic for Psychosomatic Medicine, Psychotherapy und Medical Psychology, Technical University of Munich, München, Germany; 2 Department for Surgery, Psychosocial Care Unit, University Hospital Heidelberg, Heidelberg, Germany

**Keywords:** cancer, psychological distress, psychosocial aspects, psycho-oncology

## Abstract

The purpose of this study was to assess the psychological distress of cancer patients in a disease-specific manner as well as the demographic and medical variables that have an impact on the distress. Psychological distress was assessed with the Questionnaire on Stress in Cancer Patients revised version, which has been developed and psychometrically evaluated in Germany. It consists of items about 23 cancer-specific stress situations, which have to be answered in terms of relevance and amount of distress. A heterogeneous sample of 1721 cancer in- and outpatients was assessed. For the total group, the most important distress is the fear of disease progression. We consider between 23.4% (ca. of the upper gastrointestinal tract) and 40.9% (breast cancer patients) as highly distressed. The most distressed diagnostic subgroups are patients with soft tissue tumours and breast cancer patients. There are no global (general) stress factors, as the relevant demographic and medical ‘risk factors’ varied between the diagnostic subgroups. Cancer-specific distress questionnaires give a more precise insight into patients' experience than general or psychiatric questionnaires. They are not only used in large screening studies but also in routine medicine, particularly when the objective is to identify patients to whom psycho-oncological support is to be given.

There is much research demonstrating that cancer patients suffer from a significant amount of psychological distress. The assessment of psychological distress does not only play a role within epidemiological/basic research but also increasingly within routine clinical practice.

Usually ‘psychological distress’ is interpreted and assessed as psychiatric morbidity or prevalence of psychiatric disorders, especially anxiety disorders and depression. The prevalence rate for psychiatric disorders in general among cancer patients varies between 5 and 50%, for depressive disorders between 0 and 46% and for anxiety disorders between 1 and 49% (Derogatis *et al*, 1983; van't Spijker *et al*, 1997; Sellick and Crooks, 1999; Härter *et al*, 2001; Zabora *et al*, 2001a). The most frequently used screening instruments on which these findings are based are the Profile of Mood States (POMS; McNair *et al*, 1992), the Symptom Checklist (SCL-90-R; Derogatis, 1977) or its short form, the Brief Symptom Inventory (BSI; Derogatis, 1993), the State Trait Anxiety Inventory (STAI; Spielberger *et al*, 1970), the Hospital Anxiety and Depression Scale (HADS; Moorey *et al*, 1991), the General Health Questionnaire (GHQ; Goldberg and Williams, 1988) and the Sickness Impact Profile (SIP; Bergner *et al*, 1981).

These questionnaires are either psychiatric or unspecific instruments. They have not been developed for cancer patients. (In the field of quality of life research, cancer-specific questionnaires are used, for example, the EORTC Quality of Life Questionnaire QLQ C30 (Aaronson, 1991) or the Functional Assessment of Cancer Therapy, FACT (Cella *et al*, 1993)).

The application of such tests within oncology does not give adequate insight into the subjective health of tumour patients.

On the one hand, tumour patients are asked questions that have little relevance for most of them (psychiatric symptom items) and on the other hand, the specific needs and distress of such chronic physically ill are only insufficiently recognised, for example, real, non-neurotic fears, communication disorders, feelings of physical imperfection, somatic and social consequences as a result of treatment (Lee-Jones *et al*, 1997; Stark *et al*, 2002).

This makes for a distorted picture, for instance, in patients whose psychological findings are not consistent with those of a psychiatric disorder but nevertheless exhibit signs of high subjective distress and require psychosocial support.

In general, disease-specific questionnaires deliver results that more adequately mirror the experiences of the patient; they are more clinically relevant, because they more clearly pinpoint (psychooncological) treatment consequences (Thewes *et al*, 2004).

We present findings from a large German cross-sectional screening study that are based on a measuring instrument specifically developed to determine psychosocial stress in cancer patients, the Questionnaire on Stress in Cancer Patients revised version (QSC-R23; Herschbach *et al*, 2003). The purpose of this study was to assess cancer-specific stress situations and to identify demographic and medical variables that are related to the psychological distress.

## MATERIALS AND METHODS

### Subjects and procedure

The total sample comprises subsamples that come from different regions within Germany and have been treated at different stages of treatment in various treatment settings, for example, public hospitals, university clinics, rehabilitation clinics, outpatient clinics, in addition to a subsample of patients who have been contacted at home (follow-up investigation). All patients were informed of the objectives of the study and that participation was voluntary. After being given instructions, they then completed the questionnaire on their own.

### Data collection

Psychological distress was assessed with the ‘Questionnaire on Stress in Cancer Patients revised version’ QSC-R23 (Herschbach *et al*, 2003; see attachment). The QSC is a disease-specific questionnaire to assess psychosocial stress in cancer patients (all diagnoses and treatment settings). It contains 23 items that describe potential everyday stress in all areas of life in detail and in everyday language. Each problem has to be answered twice: does it apply to the test person at present and – if it does apply – to what extent does this problem cause distress? The range of the response categories varies between 0 (=the problem does not apply to me) and 5 (=the problem applies to me and is a very big problem). The items are grouped into five homogeneous scales: psychosomatic complaints, fears, information deficits, everyday life restrictions and social strains.

The development of the QSC took place in Germany and was carried out in several phases, including detailed interviews and preliminary test versions (Herschbach *et al*, 1985; Herschbach and Henrich, 1987) and psychometric evaluations (Herschbach *et al*, 2003). The construct validity has been demonstrated by correlation analysis with diverse psychological tests such as HADS depression (*r*=0.75, *n*=578), HADS anxiety (*r*=0.73, *n*=579) or SCL-90-R (*r*=0.76, *n*=171). The discriminant validity and the sensitivity to change has also been demonstrated (Herschbach *et al*, 2003). The reliability has been analysed via Cronbach's alpha, which is 0.89 (*n*=1349) for the total score.

### Statistical analyses

Statistical evaluation was performed using the Statistical Package for Social Sciences program (SPSS; Version 10.0). Group differences were tested with *t*-tests or *F*-tests. To verify the cutoff point >1.5 of QSC-R23 total score, a receiver–operator characteristic curve analysis was performed with anxiety and depression as categorical variables measured with the Hospital Anxiety and Depression Scale (recommended cutoff values >11; Moorey *et al*, 1991), a questionnaire which is widely used in oncology. We used the Youden index (sensitivity+specificity−1) as a measure of accuracy (Youden, 1950).

To identify the proportion of ‘risk patients’ in different subgroups within each diagnostic group, we used CHAID (‘CHi-squared Automatic Interaction Detector’), which is part of AnswerTree (SPSS). The CHAID is a multivariate exploratory technique and a nonparametric alternative to the hierarchical regression approach. In contrast to the regression approach, CHAID has no restrictions regarding the measurement level or the frequency distribution of the variables. It is concerned with the discovery and specification of population subgroups that differ in their probability of an event (using chi square tests). This event is, in our case, the association of the patients to the group of ‘highly distressed’ or ‘risk patients’.

In the first step, CHAID examines the categories of each predictor for significance with respect to the dependent variable in the total sample. If possible and useful, categories are merged with others. Continuous predictors (such as age or illness duration) are analysed for one or more single cutoff values to split the sample. Finally, the most significant predictor is selected for segmenting the sample. In the next step, CHAID moves down the tree, splitting on the best predictor, and analyses each subgroup in turn. This process is continued until there is no significant predictor (*P*>0.010), or the specified stopping rules are fulfilled (e.g. minimum number of cases in a subgroup=10).

## RESULTS

### Sample

The total sample comprises 1721 cancer patients (cf. Table 1

**Table 1:** Sample description

	***n***	**%**
Age(years)		
< 40	231	13.7
40–49	292	17.3
50–59	507	30.0
60–69	397	23.5
> 69	264	15.6
		
Gender		
Female	958	56.1
Male	750	43.9
		
Partnership		
With partner	1107	75.3
Without partner	363	24.7
		
Cancer diagnoses		
Breast	394	22.9
Haematological neoplasias	326	18.9
Gynaecological carcinomas	236	13.7
Upper gastrointestinal tract	145	8.4
Respiratory tract	124	7.2
ENT^a^ carcinomas	117	6.8
Lower gastrointestinal tract	108	6.3
Male genitourinary tract	99	5.8
Urinary tract	45	2.6
Brain tumours	42	2.4
Soft tissue tumours	32	1.9
Thyroid carcinomas	14	0.8
CUP^b^, other diagnoses	21	1.2
Missing diagnoses	18	1.0
		
Metastases		
Yes	407	29.2
No	986	70.8
		
Illness duration		
< 6 months	677	46.9
6 months–2 years	353	24.5
>2 years–5 years	247	17.1
>5 years	166	11.5
		
Treatment setting		
Inpatient clinic	1090	63.4
Rehabilitation center	425	24.7
Outpatient settings	11	0.5
No actual treatment	194	11.3

a Ear, nose and throat.

b Cancer with unknown primary tumour.

). Most patients are between 50 and 69 years old; 56.1% are female.

There is a wide range of 12 different diagnostic subgroups. The most frequent were breast cancer (22.9%), haematological neoplasias (18.9%) and gynaecological cancers (13.7%). A total of 21 patients had cancers of unknown primary tumour (CUP) or very rare diagnoses and 18 patients were without diagnosis (missing). In all, 1090 patients were recruited in hospitals, 425 in rehabilitation centres and 11 in outpatient settings. A total of 194 persons were not undergoing treatment at the time of assessment; they were contacted by mail during a follow-up study.

### Psychosocial distress

The results are presented in the following order. First of all, we look at the stress scores (items and scales of the QSC-R23) for the total sample. Then, we present the variables that have a significant impact upon the total stress score of the QSC-R23 in the total sample (univariate subgroup comparisons) and finally we look at the relevant variables for each diagnostic category separately (multivariate analyses).

The ranked mean stress scores of the 23 items of the QSC-R23 questionnaire (see the appendix) for the total sample are presented in Table 2

**Table 2:** Stress scores of the single items of the QSC-R23

	***M***	**s.d.**	**% 4/5**
Being afraid of disease progression	2.41	1.80	32.2
Feeling tired and weak	2.06	1.68	22.3
Being afraid of having to go to the hospital again	1.92	1.78	23.2
Not being able to follow one's hobbies	1.91	1.79	24.0
Being afraid of developing pain	1.77	1.73	20.9
Having trouble sleeping	1.76	1.74	20.7
Feeling often tense and nervous	1.75	1.58	16.2
Being afraid of not being able to work anymore	1.66	1.83	21.3
Going out less	1.46	1.70	17.2
Having sex less frequently	1.45	1.71	16.0
Feeling physically imperfect	1.34	1.66	15.0
Suffering pain due to surgery	1.19	1.49	9.8
Suffering pain due to unknown causes	1.10	1.52	0.1
Feeling not adequately informed about social support	0.98	1.51	10.8
Feeling not well informed about illness/treatment	0.84	1.37	7.3
Body care has become difficult	0.80	1.37	7.8
Difficulty for partner to empathise my situation	0.70	1.27	5.6
Having too few opportunities to talk about emotional problems	0.69	1.29	6.5
Different information from different doctors	0.68	1.31	6.5
Feeling unconfident in relationships with other people	0.66	1.21	4.9
Having the feeling of being less value for other people	0.57	1.19	5.3
Difficulty in talking with the family	0.55	1.19	5.3
Other people react inconsiderately/unsympathetically	0.55	1.13	4.3

a Questionnaire on Stress in Cancer Patients – revised version.

(first column). A very broad spectrum of problems and burdens is mapped here. It contains physical and psychological complaints, fears, social problems, information deficits, etc.

The third column shows the percentage of patients who used the highest response categories (strongly/very strongly distressed). As can be seen, the most frequent problems are the fear of disease progression (32.2%), of not being able to follow one's hobbies (24%) and the fear of having to go to hospital again (23.2%).

The mean scores for the five subscales are: fears *M*=1.94 (s.d.=1.40), psychosomatic complaints *M*=1.57 (s.d.=1.13), everyday life restrictions *M*=1.40 (s.d.= 1.16), information deficits *M*=0.80 (s.d.= 0.96) and social strains *M*=0.61 (s.d.= 0.84). The total score is M=1.26 (s.d.=0.83).

### Univariate subgroup comparisons

In order to find out which medical and sociodemographic factors have an impact on the distress of the patients (QSC-R23 total score), we looked at the variables age, gender, partnership, diagnoses, metastases (yes/no), illness duration and treatment setting. Table 3

**Table 3:** QSC-R23 total score for subgroups of cancer patients

	***M***	**s.d.**	***P***
Age			
< 40	1.25	0.86	
40–49	1.36	0.89	<0.001
50–59	1.34	0.81	
60–69	1.20	0.79	
>69	1.05	0.77	
			
Gender			
Female	1.32	0.85	<0.001
Male	1.18	0.79	
			
Partnership			
With partner	1.22	0.80	0.823
Without partner	1.23	0.90	
			
Metastases			
Yes	1.35	0.83	0.002
No	1.21	0.80	
			
Illness duration			
< 6 months	1.15	0.78	
6 months–2 years	1.34	0.87	<0.001
>2 years–5 years	1.34	0.83	
> 5 years	1.18	0.83	
			
Treatment setting			
Inpatient clinic	1.22	0.81	
Rehabilitation center	1.39	0.82	0.002
Outpatient settings	1.45	0.87	
No actual treatment	1.17	0.91	

and Figure 1

**Figure 1:**
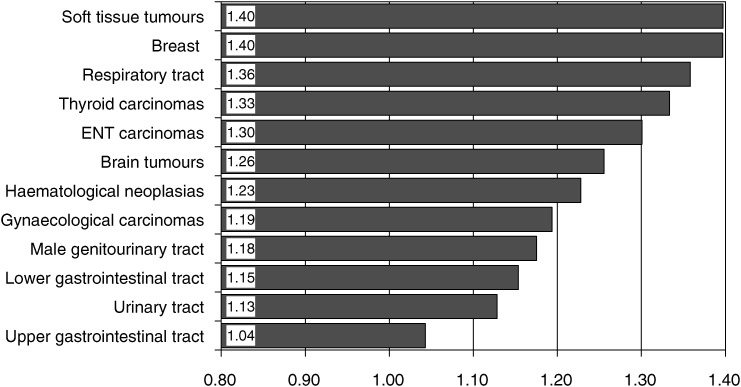
QSC-R23 total scores for the diagnostic subgroups.

show the corresponding subgroup comparisons.

All the variables, with the exception of partnership, have a significant impact on the patient's distress.

The results of the variable diagnoses are presented in Figure 1. Patients with soft tissue tumours and breast cancer patients have the highest stress scores; patients with the lowest scores are those with tumours of the GI tract and with tumours of the urinary tract. The subgroup differences are significant (*P*=0.002).

### Multivariate subgroup comparisons

The last step in our evaluation includes multivariate subgroup comparisons. The statistical procedure we used here is CHAID. The dependent variable in CHAID is the association of the patients to the dichotomised QSC-R23 total score. We used a cutoff point at the 66th percentile, that is, all patients with a QSC-R23 total score >1.5 are considered as ‘highly distressed’ or ‘risk patients’.

The reason for choosing this cutoff point is that in most of the relevant studies, the proportion of highly stressed cancer patients varies between 25 und 30% (van't Spijker *et al*, 1997; Weis and Koch, 1998; Sellick and Crooks, 1999; Härter *et al*, 2001; Zabora *et al*, 2001a). Additionally, we made an attempt to validate this cutoff point. We used data from an independent QSC-R23 data file (*n*=596), which also contains data from the HADS. The ROC analyses revealed an optimum cutoff >1.69 for the QSC-R23 total score (sensitivity 88%, specificity 72%, Youden index 0.599) to classify depression and a cutoff >1.64 (sensitivity 90%, specificity 71%, Youden index 0.611) to classify anxiety. These values are close to 1.5 and justify the use of this cutoff point.

Six medical or sociodemographic variables were analysed as potential predictors of the QSC-R23 cutoff score for the ‘risk group’: age, gender, partnership, metastases, illness duration and treatment setting. We looked at the impact of medical or sociodemographic factors separately within each diagnostic subgroup that included at least 99 patients: breast cancer (*n*=394); gynaecological cancer (*n*=236); ENT carcinomas (*n*=117); haematological neoplasias (*n*=326), upper GI tract (*n*=145), lower GI tract (*n*=108), respiratory tract (*n*=412) and male genitourinary tract (*n*=99).

**Figure 2:**
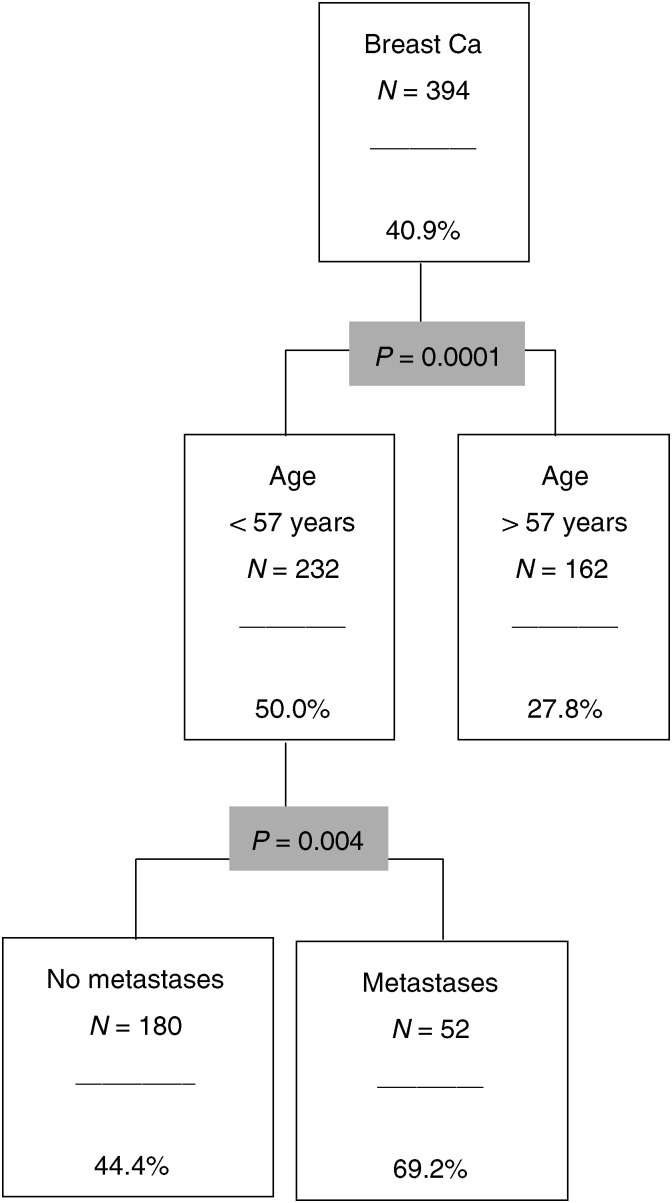
The CHAID analysis for breast cancer.

The CHAID results are presented for each of those diagnostic groups as figures (Figures 2

, 3

**Figure 3:**
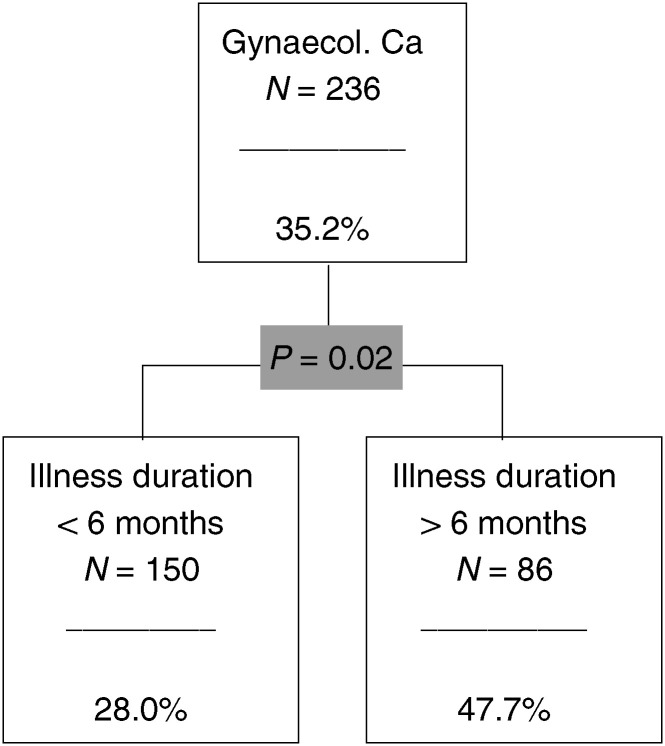
The CHAID analysis for gynaecological cancers.

, 4

**Figure 4:**
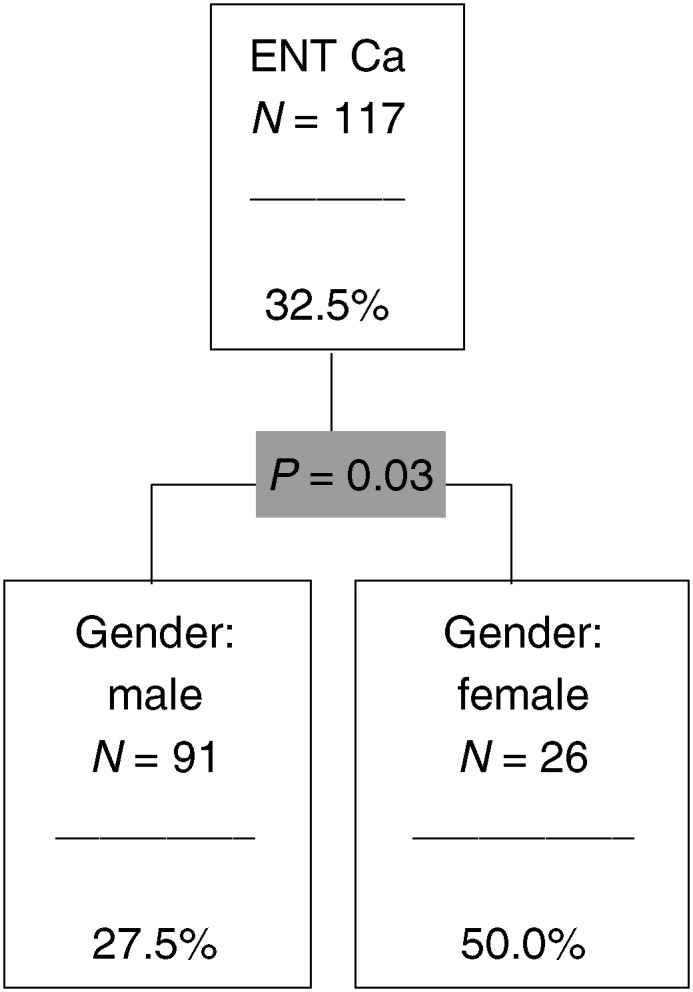
The CHAID analysis for ENT (ear, nose and throat) cancer.

, 5

**Figure 5:**
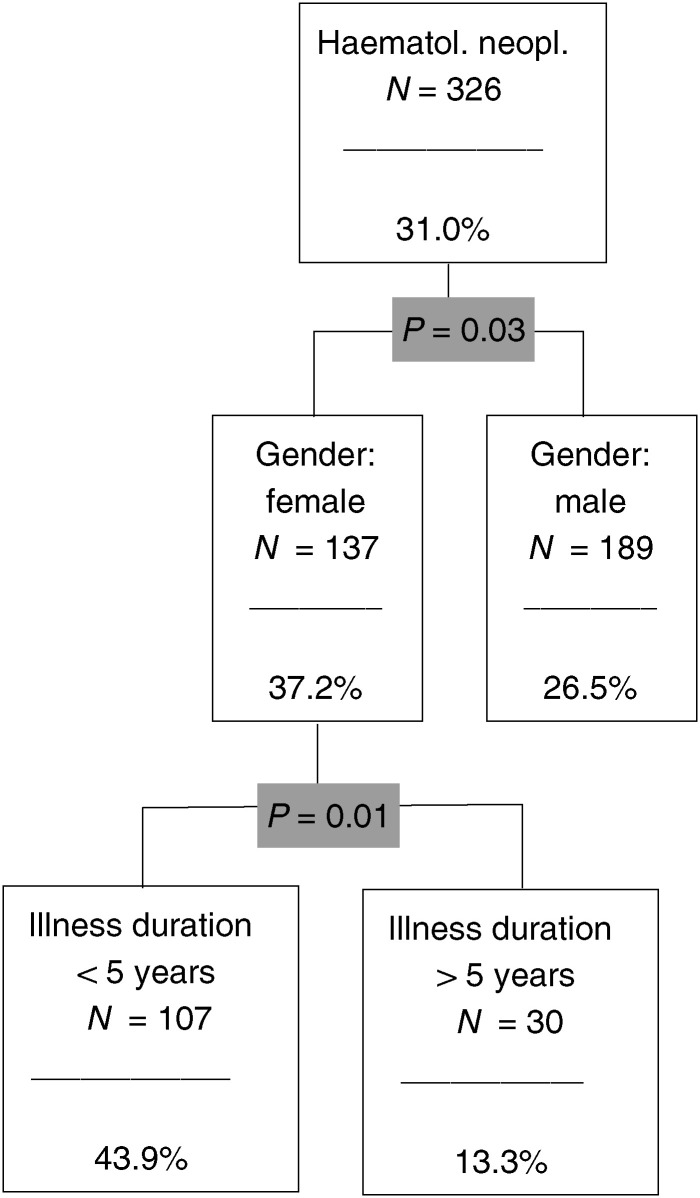
The CHAID analysis for haematological nedoplasias.

, 6

**Figure 6:**
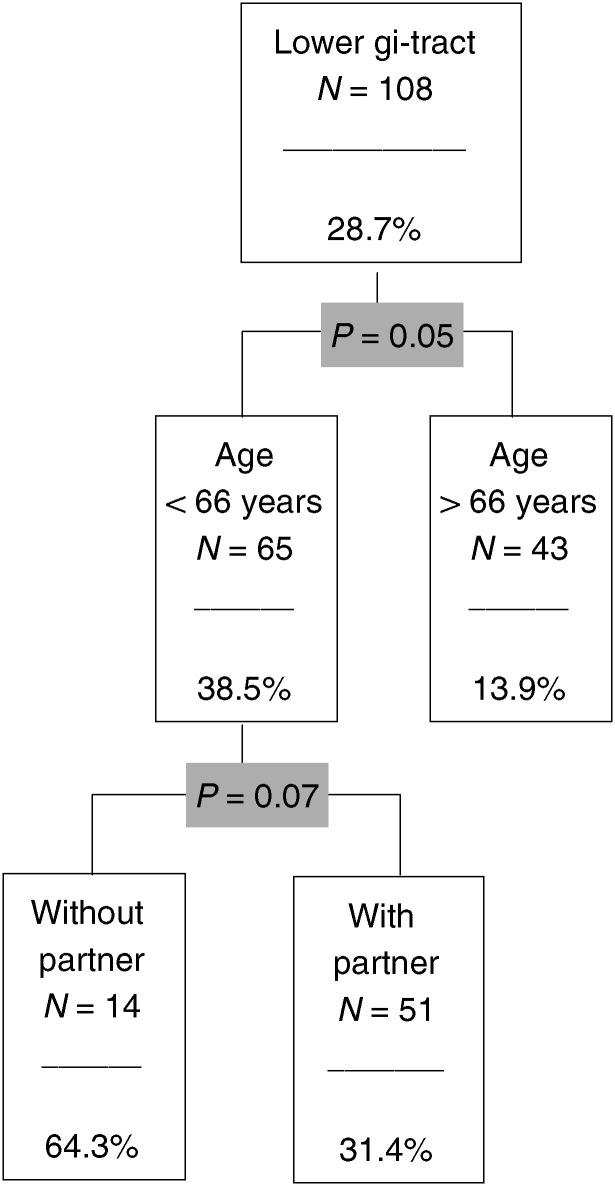
The CHAID analysis for the lower gastrointestinal tract.

and 7

**Figure 7:**
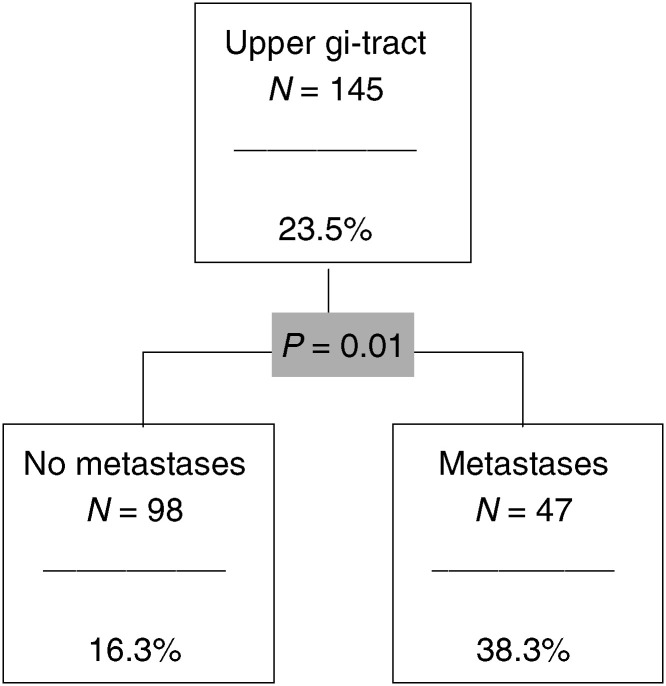
The CHAID analysis for the upper gastrointestinal tract.

). The figures show the relevant predictors and categories (*P*-value included) and the number and percentage of ‘risk patients’ within each subgroup. The *P-*values <0.10 were regarded as significant.

### Breast cancer

In total, 40.9% of the 394 breast cancer patients belong to the risk group (Figure 2). The most important predictor is age. Patients under 57 years of age are more stressed than the older patients. The most stressed subsample is made up of younger patients with metastases. The proportion of risk patients here is 69.2%.

### Gynaecological cancer

A total of 236 patients have gynaecological cancer; 35.2% are in the risk group (Figure 3). The only significant predictor variable is illness duration. The proportion of risk patients is highest in the subgroup of patients with an illness duration of more than 6 months (47.7%).

### Ear, nose and throat cancer

In all, 32.5% of all ENT patients belong to the risk group. This proportion goes up to 50.0% in the subgroup of female patients.

### Haematological neoplasias

Here, we find two significant predictors: gender and illness duration (Figure 5). The most stressed subgroup is made up of female patients with an illness duration of less than 5 years. The risk proportion here is 43.9% compared to 31.0 % in the total group.

### Cancer of the lower gastrointestinal tract

This diagnostic subgroup consists of 108 patients; the risk proportion is 28.7% (Figure 6). The most important predictor is age. Patients under 66 years belong to the group of risk patients. The second predictor is the partner situation (*P*=0.07). The small group of younger patients without a partner has a high proportion of risk patients (64.3%).

### Cancer of the upper gastrointestinal tract

The only relevant predictor here is metastases (Figure 7). In total, 23.5% of the whole group (*n*=145) belong to the risk group. The corresponding number of patients with metastases is 38.3%.

### Cancer of the respiratory tract

Among the 412 patients with cancer of the respiratory tract, 37.9 % belong to the risk group. No significant predictor could be found in this diagnostic subgroup.

### Male genitourinary tract

A total of 31.3% of the 99 patients with cancer of the male genitourinary tract are in the risk group. Here again, no significant predictor was found.

Summarising the CHAID results, we found that the percentage of risk patients varies among the diagnostic subgroups between 40.9% in the breast cancer group and 23.5% in the upper gastrointestinal cancer group. The predictors also vary between the diagnostic subgroups; thus we found that there are no general risk factors for psychological distress. The younger breast cancer patients with metastases form the most stressed single subgroup.

## DISCUSSION

This is a large cross-sectional study about the psychological morbidity of cancer patients assessed with a disease-specific psychometrically evaluated questionnaire. We looked at the distress profile, compared the diagnostic subgroups and analysed risk factors for high distress within the diagnostic subgroups.

Our results are based upon a large and heterogeneous sample, which included diverse diagnoses, disease stages and treatment settings. For the total group of 1721 patients, the most important single distress is the fear of disease progression (Herschbach *et al*, 2004). There is a significant impact upon the total stress score for the variables age, gender, metastases, illness duration, treatment setting and diagnoses. Patients with soft tissue tumours and breast cancer patients have the highest stress scores. The proportion of patients within the diagnostic subgroups, which we considered highly distressed or as risk groups, varies between 23.5% (cancer of the upper gi tract) and 40.9% (breast cancer).

When we look at the determinants for psychological distress within each diagnostic category separately, we see a very heterogeneous picture.

One important sociodemographic variable is gender. Generally (not only in oncology), we see that females demonstrate higher stress scores in psychological tests than males. This is also true in our total sample as well as for the diagnostic subgroups of patients with haematological neoplasias and ENT cancer. For the breast and gynaecological cancer patients, the variables diagnosis and gender are confounded. This is probably one reason for the high stress scores of these patients. That especially for the younger breast cancer patients the stage of disease (metastases) is an important risk factor is very plausible.

In general, metastases are of less relevance than to be expected. For six of the eight subgroups it does not play a major role in subjective stress.

The illness duration is an important risk factor for the patients with gynaecological cancers and haematological neoplasias. For the first group, the critical time frame is the first 6 months after diagnosis. This is the time that might here be considered a critical marker for the further prognosis, whereas the haematological patients with much longer treatment durations seem to orientate themselves more to the conventional 5-year survival rate criteria.

Although we can find plausible interpretations for the relevance of single risk factors within some diagnostic subgroups, it seems difficult to explain why the same factors do not play the same role within other subgroups (e.g. relevance of age for patients with cancer of the lower gastrointestinal tract but not for patients with cancer of the upper gastrointestinal tract). We probably need to take into account complex interaction patterns between variables. Also we would need larger and more homogeneous samples for each diagnostic subgroup to have a chance to clarify these interaction patterns.

There is a second problem with our data. Our list of potential stress determinants is certainly incomplete. In order to match the data from the different centres and for practical reasons, we used only variables that could be assessed by the patients themselves. All the objective factors that are concerned with the cancer prognosis could therefore not be included.

We also did not consider the actual treatment situation. This is a variable that might have an influence upon the subjective distress, but is a very complex one. Most of the patients were undergoing treatment at the time of our assessment. The treatment was surgery, radiotherapy, chemotherapy or a combination of these, for the first time or repeatedly. This variable again is confounded with the disease stage and also the diagnosis.

For those reasons, the results may be considered preliminary. Nevertheless, in our opinion, the differentiation of risk factors according to diagnostic subgroups using the same cancer-specific distress instrument has proved to be worthwhile. This does not mean that global or psychiatric scales do not have any relevance in oncology; they play a role in comparison or epidemiological studies. Also, they are useful in identifying psychiatric diseases within samples of cancer patients.

For further research, adequate sample sizes and better controlled risk factor variables are suggested.

It is difficult to compare our results with the literature (e.g. Härter *et al*, 2001; van't Spijker *et al*, 1997; Zabora *et al*, 2001a) because we used a cancer-specific questionnaire rather than global or psychiatric measures. Our data are in line with Zabora *et al*, who found the highest stress scores for the BSI scales Anxiety and Somatization, and they also found that between 29.6% (gynaecological cancers) and 43.45% (lung cancer) of the patients could be considered severely stressed. Härter *et al* found that 20% of their patients had pathological scores for anxiety, and 17% for depression.

If we look at the potential risk factors for severe distress (or psychopathological test scores), we again find a rather inconsistent picture. In some studies, the diagnostic subgroups differ (as in our study), in others they do not (van't Spijker *et al*, 1997). Usually female patients and younger patients suffer more stress than male or older patients, as in our study and as also shown in Härter's and Zabora's study. We also found that metastases play an important role, but Härter *et al* did not. In our study, it was the outpatients who were more stressed, in Härter's study the in-patients were the more stressed subgroup.

The reasons for such disappointing heterogeneity are presumably manifold. One of the factors certainly is the different questionnaires and the psychodiagnostic criteria.

The results of our study can naturally only serve as a reference when it comes to clinical care. Here, it is especially important that each individual patient to be given psycho-oncological support is identified correctly and in time. This objective is increasingly seen as a part of comprehensive oncological care (Weller, 2004). This is reflected, for instance, in the Guidelines for psychosocial care in Canada (‘Psychosocial service needs of patients and families are assessed systematically using appropriate tools’ (Canadian Cancer Society).), Australia, USA (‘All patients should be screened for distress at their initial visit, at appropriate intervals, and as clinically indicated’ (American Society of Clinical Oncology).) or Germany (Carlson and Bultz, 2003; Mehnert *et al*, 2003).

In the meantime, there are several Cancer Centres endeavouring to implement these guidelines in their clinical care as part of distress screening programmes, for example, the Memorial Sloan-Kettering Cancer Centre in New York (Roth *et al*, 1998), the Sidney Kimmel Comprehensive Cancer Centre at Johns Hopkins in Baltimore (Zabora *et al*, 2001b), the Royal Newcastle Hospital in Newcastle, New South Wales, Australia (Bonevski *et al*, 2000), the Tom Baker Cancer Centre in Calgary, Canada (Carlson and Bultz, 2003) or the Clinical Cancer Centre, University Clinic Rechts der Isar in Munich (Heussner *et al*, 2004). The use of electronic questionnaire versions (e.g. tablet PCs or touch screen computers) has proved to be feasible and useful in practice (Detmar and Aaronson, 1998; Velikova *et al*, 1999; Cull *et al*, 2001). We would suggest that unspecific psychopathology questionnaires should not be used for this purpose, but rather questionnaires that are relevant to the specific experiences of cancer patients and, therefore, are of greater clinical relevancy.

## Appendix

The questionnaire is provided in Table A1

**Table A1:**

.
